# Social and clinical impact of COVID-19 on patients with fibrodysplasia ossificans progressiva

**DOI:** 10.1186/s13023-022-02246-4

**Published:** 2022-03-04

**Authors:** Samuel Kou, Sammi Kile, Sai Samhith Kambampati, Evelyn C. Brady, Hayley Wallace, Carlos M. De Sousa, Kin Cheung, Lauren Dickey, Kelly L. Wentworth, Edward C. Hsiao

**Affiliations:** 1 Division of Endocrinology and Metabolism, The UCSF Metabolic Bone Clinic, University of California-San Francisco, 513 Parnassus Ave, San Francisco, CA USA; 2 International FOP Association (IFOPA), North Kansas City, MO USA; 3 BioSAS Consulting, Inc., Wellesley, MA USA; 4 Division of Endocrinology and Metabolism, Zuckerberg San Francisco General Hospital, San Francisco, CA USA; 5 The Institute for Human Genetics, The Program in Craniofacial Biology, and the Robert L. Kroc Chair in Rheumatic and Connective Tissue Diseases III, University of California-San Francisco, San Francisco, CA USA

**Keywords:** Fibrodysplasia ossificans progressiva (FOP), COVID-19, SARS-CoV-2, FOP Connection Registry, Heterotopic ossification (HO), Vaccination

## Abstract

**Background:**

COVID-19, caused by the SARS-CoV-2 virus, is a severe inflammatory condition. Patients with pre-existing conditions including diabetes, hypertension, and cardiovascular disease are at particularly high risk of complications. Fibrodysplasia ossificans progressiva (FOP) is an ultra-rare and debilitating genetic disorder that is characterized by a pro-inflammatory state, which leads to progressive heterotopic ossification and complications after trauma, including intramuscular vaccinations. To better understand the impact of COVID-19 on patients with FOP, we first examined the social impact of the pandemic using data from the FOP Registry managed by the International FOP Association. We also identified patients with FOP who were exposed to or contracted the SARS-CoV-2 virus, or who received a COVID-19 vaccine, to investigate if patients with FOP were at increased risks of complications from SARS-CoV2 exposure or vaccination.

**Results:**

Data from 326 individuals in 69 countries in the International FOP Association FOP Connection Registry were examined using patient-reported outcomes measurement information system (PROMIS) global health scale scores. Twenty-six (28.9%) participants aged ≥ 15 years old rated their satisfaction with their social activities and relationships as poor in 2020, which was an increase from 18 (18.9%) in 2019, prior to the SARS-CoV-2 outbreak. Similar trends were noted for physical and mental health in the pediatric population. Frequency of physician visits was not changed, but a larger portion of patients reported missing dental visits in 2020 compared with 2019 (31.5% vs. 41.7%). A second cohort with 32 subjects was tracked after SARS-CoV-2 exposure or vaccination. Ten subjects were positively diagnosed with COVID-19, 15 received a COVID-19 vaccine, and seven had high-risk SARS-CoV-2 exposure but either did not have a confirmed clinical diagnosis or tested negative. Subjects who tested positive for the virus showed no major complications or increased FOP disease activity, though our sample size is very limited. Among the 15 subjects who received a COVID-19 vaccine, using the International Clinical Council on FOP guidelines for prophylaxis with ibuprofen or acetaminophen, only one person experienced flare-like activity at the injection site.

**Conclusions:**

Patients with FOP showed a significant decrease in social activities that was reflective of the isolation and mobility changes in this debilitated population. In our limited cohort, the majority of the patients with FOP who tested positive for COVID-19 showed no major complications. Also, although limited in sample size, the majority of patients who received a COVID-19 vaccination and followed guidelines from the FOP International Clinical Council tolerated vaccination well. Only one person experiencing flare activity following their injection. Thus, the risks and benefits of COVID-19 vaccination needs to be discussed carefully so as to support informed decisions.

## Introduction

Coronaviruses (CoVs) are a class of enveloped, single-stranded RNA viruses found globally in humans [1]. While coronavirus infections in humans are typically mild, several previous coronavirus outbreaks have been associated with significant morbidity and mortality. The first cases of severe acute respiratory syndrome coronavirus 2 (SARS-CoV-2) were reported in December 2019 [2]. The ensuing coronavirus (COVID-19) pandemic has spread worldwide, with over 177 million reported cases as of June 2021 [3]. Declared a Public Health Emergency of International Concern by WHO (World Health Organization) on January 30^th^, 2020, the COVID-19 outbreak has resulted in significantly worse outcomes for patients suffering from pre-existing conditions including diabetes, hypertension, and cardiovascular disease [4]. In addition, the COVID-19 outbreak has led to increased isolation and loss of in-person social interactions, which can have particularly negative impacts on patients with debilitating health conditions [5].

Fibrodysplasia ossificans progressiva (FOP) is a rare genetic condition characterized by progressive heterotopic ossification (HO) of tendons, ligaments, and other soft tissues within the body [6]. This ectopic formation of bone tissue is debilitating, causing chronic pain, restricted range of motion, pulmonary dysfunction, and impaired mobility. Although HO can occur spontaneously, incidents have been correlated to inflammation arising from trauma, tissue damage, viral infections [7], intramuscular diphtheria-tetanus-pertussis immunizations [8], and other traumatic stimuli [9]. In about half of cases of HO formation, individuals report episodic flare-ups, which are characterized by symptoms such as swelling, warmth, and stiffness [9].

FOP is caused by heterozygous activating mutations to the *ACVR1* gene, which encodes ACVR1 (also known as ALK2), a bone morphogenic protein (BMP) type 1 receptor [10]. The most common of these mutations is c.617G>A, p.R206H [10], which causes the receptor to misinterpret the Activin A ligand as a BMP-like molecule, leading to activation of HO formation [11]. Barruet et al. showed that individuals with FOP have a pro-inflammatory state at baseline, even in the absence of ectopic bone formation or flare-ups [12]. Specifically, elevated levels of pro-inflammatory and myeloid cytokines were observed, which may suggest a role for the *ACVR1* R206H mutation in causing this heightened immune response [13]. In FOP mouse models, abundant macrophages and mast cells were observed in developing heterotopic lesions [14], and depletion of mast cells and macrophages decreased trauma-induced HO by 50% individually and 75% combined [15]. Furthermore, Activin A is a major cytokine produced by macrophages, including FOP macrophages [13], and is a key regulator of macrophage polarization [16].

Clinically, FOP progression can be promoted by viral-like illnesses. A prior study assessed if FOP patients who had symptoms of influenza during the 2000–2001 influenza season experienced an increase in the incidence of HO-related flare-ups [7]. The FOP subjects exhibited a minimum of a three-fold increased risk of flare-ups when exposed to viral illness compared with before viral exposure, indicating that influenza-like viral illnesses are associated with flare-ups in FOP patients [7].

The severe respiratory compromise from thoracic insufficiency induced by HO formation in the chest wall, as well as the high risk of complications from intubation, put patients with FOP at particularly high risk of adverse outcomes in the event of severe COVID-19 complications [17]. The thoracic insufficiency syndrome is a result of multiple factors including costovertebral malformations, ankylosis of the costovertebral joints, and ossification of the intercoastal and paravertebral muscles [18]. This can lead to impaired pulmonary function and can also cause pneumonia and right-sided congestive heart failure [18]. In patients requiring respiratory care, surgical intubation procedures can give rise to complications, notably triggering of HO at the intervention site [17, 19]. Additionally, due to the prevalence of jaw mobility limitations, flare-ups, and neck malformations, patients with FOP incur significant risk for advanced respiratory management such as with intubation [17].

A study looking at serum samples of patients with severe, active COVID-19 infections found that Activin A, Activin B, and follistatin were all upregulated during the time when COVID-19 patients tended to deteriorate [20]. In a COVID-19 study defining Acute Respiratory Distress Syndrome (ARDS), cytokine storms were observed in patients with severe COVID-19 symptoms [21]. The described pathway underlying ARDS, activated by cytokines, induces the production of Activin A in skeletal muscle, resulting in skeletal muscle atrophy.

Surprisingly, a small number of patients with severe COVID-19 appear at higher risk for developing HO [22], suggesting that the pro-inflammatory state found in COVID-19 could be detrimental in patients with FOP. Aziz et al. highlighted the prevalence of shoulder HO in two female subjects (with a history of hypertension and type 2 diabetes, and hypertension, respectively) after their COVID-19 disease [22]. A few months following hospitalization, the subjects reported shoulder pain/discomfort, which was identified as ossification. The study team indicated chronic hospitalization and hypoxia as the determinant factors.

A recent case report implicated the COVID-19 vaccine as a potential trigger for myositis ossificans-related intramuscular nodule formation and internal calcifications in a 51-year-old male patient [23]. Three months following the administration of the second COVID-19 vaccine dose, the patient reported right upper arm pain and a palpable mass, without any erythema or systemic symptoms. In addition to the trauma at the injection site, the subject’s immune response to the vaccine antigen was also thought to be a causal factor for the reported pain.

In this study, we set out to answer three main questions. First, how has the COVID-19 pandemic impacted the care and mental health of patients with FOP? Second, do patients with FOP who were exposed to SARS-CoV-2 have increased FOP disease activity? And third, does vaccination against SARS-CoV-2 impact FOP flare activity?

## Methods

### The FOP connection patient registry

The social impact of the COVID-19 pandemic on patients with FOP was examined using the FOP Patient Registry sponsored by the International FOP Association (IFOPA). The FOP Registry was launched by the IFOPA in 2015 with a vision to develop a unified global registry that assembles the most comprehensive data on patients with FOP. The FOP Registry is an ongoing international, voluntary, observational resource that captures demographic and disease data directly from individuals with FOP approximately every six months via a patient portal, and clinical data from treating FOP physicians on an annual basis via a secure web-based medical portal. The design of the FOP registry is described separately [24, 25]. All participants provided consent to participate in the registry (Advarra IRB approval number: CR00263401). Participants reported the number of times they visited physicians and answered questionnaires about their social activities. Data from 2019 and 2020 were collected and compared.

To participate in the FOP Registry, patients were required to confirm their FOP diagnosis and provide informed consent via an electronic acceptance. As of March 24, 2021, 326 individuals from 69 countries were enrolled and active, representing ~ 36% of the known global FOP populations based on IFOPA estimates. Thus, this cohort is representative of the global FOP community.

### Social assessment instruments

Domain scores of the PROMIS Global Health Scale [26] were calculated to determine physical health, mental health, and social activity satisfaction in patients aged 0–14 and ≥ 15 years old based on five questions; higher scores represent a higher quality of life. Domain scores of the PROMIS questionnaire were calculated for 2019 and 2020 to evaluate scores prior to and during the COVID-19 outbreak, respectively. These scores were compared to understand the effect of the COVID-19 outbreak on FOP patients across all domains. Registry questions related to encounters with medical and dental care providers referred to the number of times the FOP patients visited a physical health, mental health, and dental health physician in both 2019 and 2020.

### COVID-19 and COVID-19 vaccination in FOP—subject recruitment

Patients with FOP who had either a known high risk exposure to the SARS-CoV-2 virus (i.e., immediate household member or close contact who tested positive), tested positive for COVID-19, or received a COVID-19 vaccine, were recruited and enrolled into the study between July 14th, 2020 and August 23rd, 2021. The study was approved by the UCSF Institutional Review Board (UCSF IRB Approval# 20-30734). After obtaining written consent, subjects with known exposure to the virus were given questionnaires covering symptoms experienced and their duration, test results, hospitalizations, complications, and any flare activity around the time of contracting the virus. Symptoms in the questionnaire included fever, chills or shakes, cough, sore throat, difficulty breathing, weakness or fatigue, unexplained muscle aches, loss of taste or smell, runny or congested nose, diarrhea, and eye redness. Subjects were followed at 2 weeks and 4 weeks to assess for any progression or changes in health status. For patients who reported their exposure retroactively, data was collected but follow-up in the aforementioned manner was forgone. Exposed subjects were considered positive if they developed COVID-19 symptoms within 2 weeks of exposure, or if they tested positive.

Subjects who received a COVID-19 vaccine were given questionnaires to collect information about the vaccine manufacturer (Pfizer-BioNTech, Moderna, etc.), injection route (subcutaneous, intramuscular, etc.) and location, side effects, any hospitalizations or complications, and any flare-like activity. All subjects were asked to follow the FOP International Clinical Council (ICC) guidelines for vaccination (summarized in Table 3, and the full document is found at ICCFOP.org). Two subjects chose to do subcutaneous vaccination instead of following the manufacturers’ recommended intramuscular route. Side effects assessed in the questionnaire included pain or soreness at the injection site, swelling at the injection site, fever, chills, tiredness/fatigue, headaches, and heterotopic ossification formation. The questionnaire was sent at least seven days post injection to allow adequate time for potential side effects to present themselves. For subjects who received a vaccine comprised of two doses, such as the Pfizer-BioNTech, subjects completed a questionnaire after each injection, and the results were aggregated. For patients who reported the same side effect after the first or second COVID-19 vaccination dose, only a single occurrence was recorded for data collection purposes.

Because FOP is a rare condition, we also collected anonymous physician data about patients with FOP who were either exposed to the virus or received the COVID-19 vaccine. Questionnaires similar to the one provided to subjects enrolled by UCSF were sent to physicians to anonymously collect the clinical information. These eight additional cases were included in our total recruitment (Fig. 4).

**Fig. 1:**
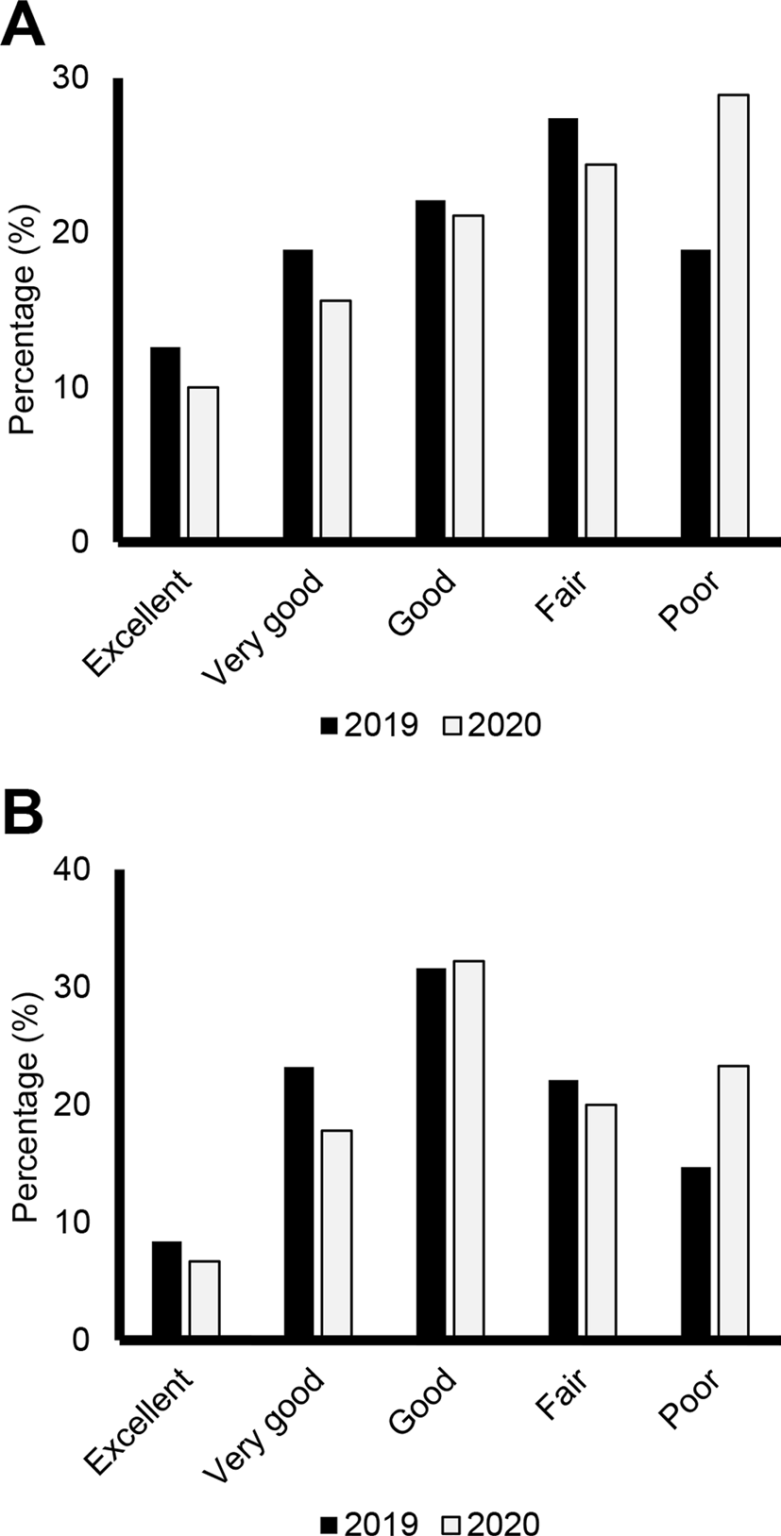
Adult PROMIS Global Health Scare Questions. Distribution of responses (Excellent, Very Good, Good, Fair, or Poor) from adult patients in the FOP Registry. **A** Level of satisfaction with social activities and relationships. **B** How well they carried out their usual social activities and roles. Dark bars represent responses from 2019 and lighter bars represent responses in 2020 (pandemic)

**Fig. 2:**
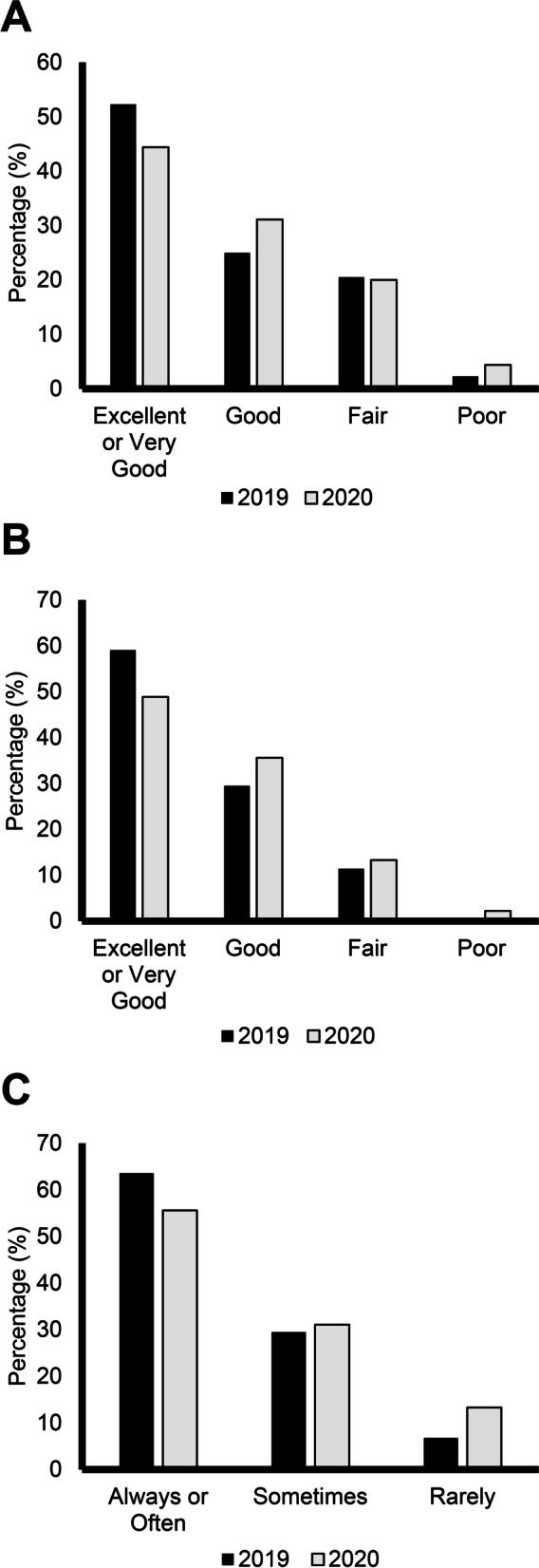
Pediatric PROMIS Global Health Scale Questions. Distribution of responses (Excellent or Very Good, Good, Fair, or Poor) from patients aged < 15 years in the FOP Registry. **A** General health level. **B** Mental health, including mood and ability to think. **C** Frequency of having fun with friends. Dark bars represent responses from 2019 and lighter bars represent responses in 2020 (pandemic)

**Fig. 3:**
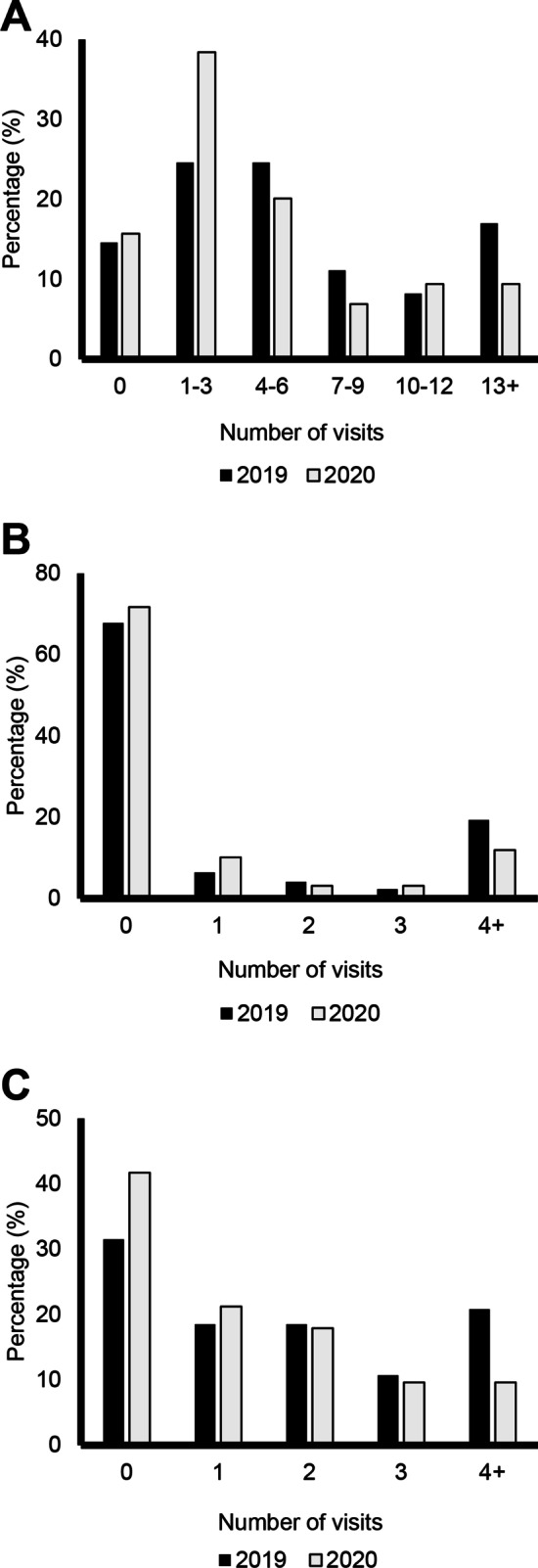
Summary of Medical Visits by Calendar Year. Distribution of the number of times patients had medical visits for: **A** Physical health. **B** Psychological/emotional health. **C** Dental care without hospitalization. Dark bars represent responses from 2019 and lighter bars represent responses in 2020 (pandemic)

**Fig. 4:**
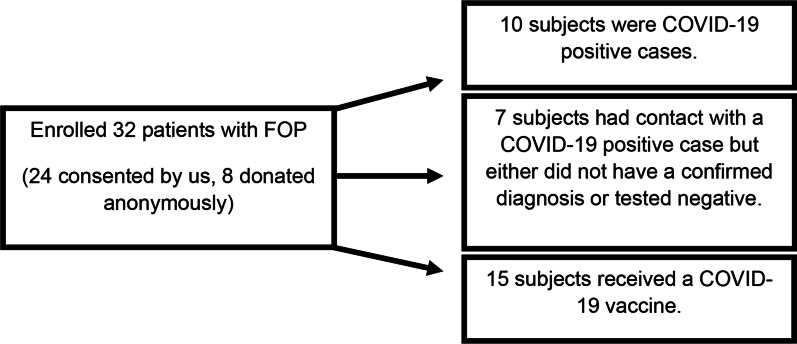
Study Enrollment: COVID-19 and SARS-CoV-2 Vaccine Study subjects. Flow chart of patients with FOP enrolled into the study (total of 32 subjects). 10 of those subjects had confirmed COVID-19 positive cases and 15 subjects received a COVID-19 vaccine. Seven subjects were enrolled based on suspected contact with another individual who tested positive for the virus, but either had no confirmed diagnosis or tested negative

**Fig. 5:**
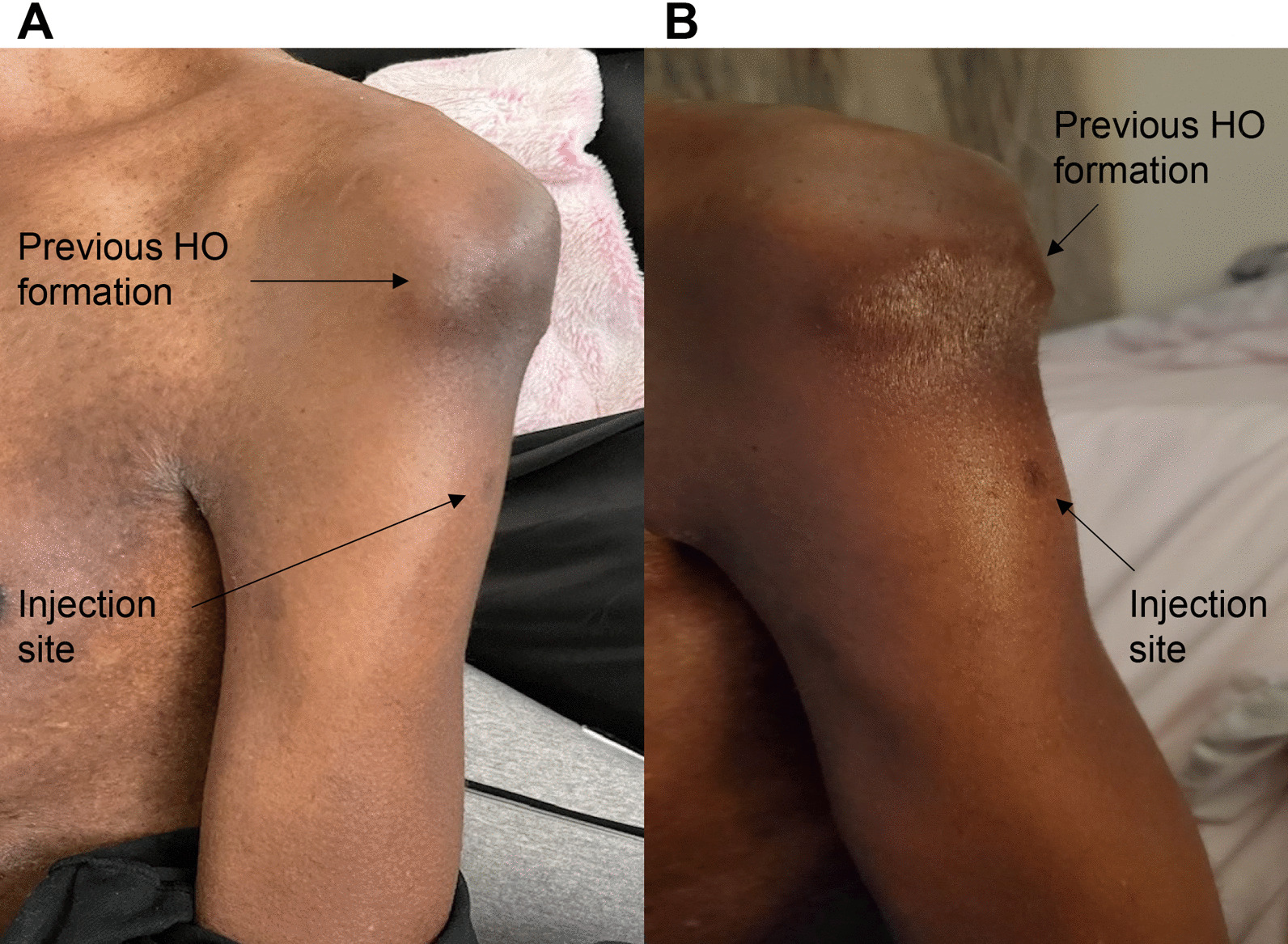
Photographs of Subject 2’s Vaccine Injection Site. A (Left) shows the injection site 20 days after the first injection. B (Right) shows the same arm a few days after the second injection. There is no change in the prior HO formation

### Statistics

Data from the PROMIS questionnaire were summarized for age groups 0–14 years and ≥ 15 years. Data related to medical and dental care providers were summarized for all age groups combined. Descriptive summaries of categorical outcomes include the number and percentage of registry participants. Percentages are based on all participants enrolled within each category and age group. Statistical comparisons for significance were not performed given the descriptive nature of the data.

## Results

### Social impact of COVID-19 on patients with FOP

#### Patient demographics and diagnosis

A total of 326 participants, predominantly from the United States, were enrolled in the FOP Registry as of March 24, 2021, the data cutoff date for this portion of the study. The mean age was 26 years and females comprised the majority (56.1%) of the cohort (Table 1). One-hundred and fifty-nine participants provided responses to the PROMIS questionnaire regarding medical and dental encounters in 2020 and 171 participants provided responses to these questions in 2019.

**Table 1:** COVID-19 ( +), Vaccination, and IFOPA- FOP Connection Registry Cohort Demographics

Characteristic	COVID-19 ( +) study subjects (*N* = 10)	Vaccination study subjects (*N* = 15)	FOP registry participants (*N* = 326)
**Current age (years)**			
Mean	31.2	31.2	26.1
Range	17.0–46.0	13.0–51.0	1.4–76.0
**Sex**			
Male	5 (50.0%)	7 (46.7%)	141 (43.3%)
Female	5 (50.0%)	8 (53.3%)	183 (56.1%)
Other			1 (0.3%)
Missing			1 (0.3%)
**Country/Region**			
United States	4 (40.0%)	10 (66.7%)	114 (35.0%)
Brazil			34 (10.4%)
Europe			30 (9.2%)
United Kingdom		2 (13.3%)	18 (5.5%)
France			15 (4.6%)
Asia			12 (3.7%)
Spain			12 (3.7%)
Switzerland		1 (6.7%)	
Serbia	1 (10.0%)		
Italy			11 (3.4%)
Canada			11 (3.4%)
India			10 (3.1%)
Poland			8 (2.5%)
Africa			7 (2.1%)
Australia			7 (2.1%)
South America			7 (2.1%)
Russia			6 (1.8%)
Chile		1 (6.7%)	5 (1.5%)
Germany			5 (1.5%)
Colombia			5 (1.5%)
North America			5 (1.5%)
South Pacific Ocean			3 (0.9%)
Missing	5 (50.0%)	1 (6.7%)	1 (0.3%)

Demographics (average age, sex, and country/ region) of the COVID-19 ( +) study subjects (10), vaccination study subjects (15), and FOP population (326) enrolled in the FOP Connection Registry. For the five anonymous subjects who were part of the COVID-19 ( +) group and the one anonymous subject who was part of the vaccination group, sex information was collected, but age and country/region data was not collected

#### PROMIS global health scale scores

Overall, 26 (28.9%) participants aged ≥ 15 years rated their satisfaction with their social activities and relationships as poor in 2020, which was an increase from 18 (18.9%) in 2019 (Fig. 1A). Adult registry participants also reported increased difficulties carrying out social activities between 2019 and 2020, as 21 (23.3%) participants answered poor in 2020 compared with 14 (14.7%) in 2019 (Fig. 1B). Similar trends were noted for a decline in general and mental health in the pediatric population. In 2019, prior to the COVID-19 outbreak, 23 (52.3%) participants ≤ 14 years rated their general health as excellent. This decreased in 2020, as 20 (44.4%) pediatric participants rated their general health as excellent (Fig. 2A). The percentage of pediatric participants rating their mental health as excellent also decreased with 26 (59.1%) in 2019 and 22 (48.9%) in 2020 rating their mental health as excellent (Fig. 2B). Social interactions were impacted by COVID-19 in the pediatric population. In 2019, 28 (63.6%) reported “always having fun with their friends,” compared to 25 (55.6%) in 2020. The number of participants reporting “rarely having fun with friends” also increased between 2019 and 2020, with three (6.8%) in 2019 and six (13.3%) in 2020 (Fig. 2C).

#### The impact of COVID-19 on medical and dental health encounters (adult and pediatric)

The number of times participants had at least one visit with a physician related to their physical health changed only slightly during the COVID-19 pandemic. In 2019, 146 (85.4%) participants reported seeing a physician one or more times, compared with 134 (84.2%) in 2020. The number of participants reporting zero visits to a physician related to their physical health remained unchanged, with 25 (14.6%) reporting no visits in 2019 and 25 (15.7%) in 2020 (Fig. 3A). Similar trends were noted in physician visits for psychological or emotional health. In 2019, 116 (67.8%) reported they did not visit a physician for a reason related to their mental health, compared with 114 (71.7%) in 2020. There was a decrease between 2019 and 2020 in the number of participants who reported seeing a physician related to their mental health at least four times; in 2019, 33 (19.3%) had at least four visits with a physician for their mental health, which decreased to 19 (11.9%) in 2020 (Fig. 3B). The number of times participants visited their dentist decreased between 2019 and 2020, and a larger percentage of FOP patients reported never visiting their dentist in 2020. In 2019, 53 (31.5%) reported never visiting their dentist, which increased to 65 (41.7%) in 2020. The number of FOP patients reporting visiting their dentist at least four times also decreased from 35 (20.8%) in 2019 to only 15 (9.6%) in 2020 (Fig. 3C).

### Clinical impact of SARS-CoV-2 infection

Patients with FOP have an increased risk of HO formation after trauma, intramuscular vaccinations, and viral infections. Although the COVID-19 pandemic had a significant social impact on patients with FOP, we also wanted to understand how patients with FOP fared after SARS-CoV-2 virus infection or COVID-19 vaccination. We identified 25 patients with FOP who either had a confirmed COVID-19 diagnosis by positive testing, or received a vaccine (Fig. 4).

Of these 25 subjects, 10 were confirmed to have contracted the virus. The mean age for the COVID-19 (+) group was 31.2 years with an even split between males and females (Table 1). Infected patients were directed to follow their local ordinances, and continued with their usual medical care through their local providers. Table 2 shows the reported numbers and frequencies of different symptoms that were present at any time during infection, quarantine, or recovery. Among the 10 subjects, only one reported the need for hospitalization, and two (including Subject 1, described below) reported flare activity around the time of COVID-19 symptoms.

**Table 2:** COVID-19 ( +) Patient-Reported Symptoms/Outcomes (*N* = 10)

Characteristic	Number of Patients	Frequency (%)
Weak/Fatigued	8	80
Loss of sense of taste or smell	7	70
Cough	6	60
Fever	5	50
Sore Throat	5	50
Headaches	3	30
Muscle Ache	3	30
Diarrhea	3	30
Difficulty Breathing	2	20
Flare symptoms	2	20
Eye redness	2	20
Chills/Shakes	1	10
Hospitalized	1	10
Runny/congested nose	1	10

List of symptoms and complications that subjects reported during COVID-19 infection. Subjects could report one or more symptoms

#### Case study 1: COVID-19 infection in a patient with FOP

Subject 1 is a 22-year-old female with the *ACVR1* R206H mutation and clinical signs of FOP. She reported a new flare in her left hip without any suspected trauma. 2–3 days later, she developed diarrhea, anosmia, and ageusia. Over the following 12 days, she reported a cough, sore throat, weakness/fatigue, runny/congested nose, and eye redness. Two weeks after reporting the flare, she tested positive for SARS-CoV-2. She experienced swelling and restricted mobility in the affected area of her hip. She was prescribed prednisone at a reduced dose of 50 mg/day for 4 days (half of the standard of care doses for flare treatment [17]) followed by a rapid taper (40 mg × 1 day, 30 mg × 1 day, 20 mg × 1 day, 10 mg × 1 day, and 5 mg × 1 day), and was advised to immediately stop the prednisone if any COVID-19 symptoms developed. The hip flare lasted approximately 7 weeks, with no major changes in the motion of the joint.

### Outcomes of patients with FOP receiving a COVID-19 vaccine

We recruited 15 patients with FOP who received a COVID-19 vaccine: 10 received the Pfizer-BioNTech vaccine, two received the Johnson & Johnson vaccine, two received the Moderna vaccine, and one received the CoronaVac vaccine. The mean age for the vaccination group was 31.2 years with females comprising 53.3% of the subjects (Table 1). Twelve subjects reported receiving their vaccine via intramuscular injection, one received the vaccine via subcutaneous injection, one patient received the first dose subcutaneously while the second dose was injected intramuscularly (Case Study 3), and one patient did not report their injection location. Subjects were asked to follow the International Clinical Council on FOP guidelines (full recommendations are at ICCFOP.org; a brief summary is in Table 3) for flare prevention after a COVID-19 vaccination, including the use of ibuprofen or acetaminophen to decrease the inflammatory symptoms post-injection.

**Table 3:** Summary of the ICC FOP Guidelines for COVID-19 Vaccine Injections

ICC-FOP Guidelines for COVID-19 Vaccine Injections Full recommendations are at ICCFOP.org	
Discuss plans with your physician and review any prior allergies or reactions Take the vaccine through its intended route (i.e.: intramuscular injection). The efficacy of taking an intramuscular vaccine subcutaneously is not known and is thus not currently recommended Take the vaccine at a location that is already fused. Avoid locations that are exposed to pressure (i.e.: back and buttocks) Be flare-free for at least 2 weeks before you receive the vaccine Use the smallest diameter needle available and, if possible, ensure that the vaccine is not injected directly next to heterotopic bone Before the vaccination, have ibuprofen, acetaminophen, and a course of prednisone available in case a flare-up arises	

Main points of the ICC FOP guidelines for patients with FOP seeking to receive a COVID-19 vaccine. Full guidelines can be found at ICCFOP.org

Table 4 shows the type and frequency of patient-reported side effects that occurred shortly following vaccination. Eleven reported experiencing pain or soreness at the injection site, six reported tiredness/fatigue, four reported having a headache, four reported swelling at the injection site, and only one subject reported having a fever. One subject reported flare-like activity (Case Study 4). No subjects reported any hospitalizations or HO formation as a result of the vaccine.

**Table 4:** COVID-19 Vaccine Patient-Reported Side Effects/Outcomes (*N* = 15)

Characteristic	Number of patients	Frequency (%)
Pain/Soreness	11	73.3
Tiredness	6	40.0
Swelling	4	26.7
Headaches	4	26.7
Fever	1	6.7
Flare symptoms	1	6.7
Chills	0	0
Hospitalized	0	0
HO Formation	0	0

List of side effects subjects reported as well as the number of subjects reporting flare activity, HO formation, or hospitalizations

#### Case Study 2: Vaccination in a patient with FOP

Subject 2 is a 47-year-old male with FOP who received both doses of the Pfizer-BioNTech vaccine intramuscularly in the left arm. He reported some pain at the injection site. Figure 5A (left) shows the injection site of Subject 2 20 days after the first injection. The patient also reported some inflammation but no sign of HO formation. Figure 5B (right) shows the same arm several days after the second injection. He again reported some pain in the arm and some swelling at the injection site. No HO formed by 2 weeks after the second injection. No steroids were taken by the patient.

#### Case Study 3: Vaccination in a patient with FOP

Subject 3 is a 51-year-old male with FOP who received the first dose of the Pfizer-BioNTech vaccine subcutaneously, against recommendations. This patient did not develop detectable anti-SARS-CoV-2 IgG antibodies in his blood after this first injection. The second injection was given in accordance with the vaccination schedule, via an intramuscular route. Subsequent testing was positive for anti-SARS-CoV-2 IgG antibodies. The patient had mild arm pain but no flare or HO formation.

#### Case Study 4: Partial vaccination in a patient with FOP

Subject 4 is a 29-year-old female with FOP who received the first dose of the Pfizer-BioNTech vaccine intramuscularly in her right deltoid. She experienced swelling, redness, and warmth at the injection site for five days. Subject 4 also experienced increased tension when extending her right arm and she was concerned about potential bone growth. She was treated with a short course of low dose (50 mg) prednisone. Her antibody titer after this first injection was positive. Subject 4 chose not to receive the second dose of the Pfizer-BioNTech vaccine due to concern over additional side effects.

## Discussion

Prior to the COVID-19 pandemic, loneliness and isolation were already major health concerns, with indications that 10–40% of people surveyed in Europe, China, and the United States were affected [27, 28]. The COVID-19 pandemic has led to significant increases in social isolation, loneliness, and mental health stress worldwide. In a study in Japan, 5.6% reported “often” or “always” feeling increased isolation during the pandemic [29]. In addition, physician visits decreased significantly, with some outpatient clinics in the USA showing a 70% decrease in patient volume [30]. A recent study from Ontario, Canada identified a 27% increase in mental health- and substance abuse-related outpatient visits [31].

The COVID-19 pandemic has disproportionately affected individuals with disabilities [32]. While these individuals have a higher risk of death resulting from COVID-19 infections, there are additional adverse effects [33], particularly from mental health issues, social isolation, and medical complications due to postponement of regular rehabilitation appointments and limited access to necessary healthcare [32]. Patients with FOP are at particularly high risk because of the additional care and devices needed to assist in the performance of everyday activities. Furthermore, as a result of flare-ups, HO can limit joint mobility, cause physical limitations, and lead to significant cardiopulmonary complications, further increasing the risk of an adverse outcome from COVID-19.

Using the IFOPA Patient Connection Registry, we identified that adult and pediatric patients with FOP had decreased participation in social activities between 2019 and 2020. This is particularly notable since mental health and pain are significant issues for patients with FOP [34]. In addition to mandated lockdowns, quarantining, and social distancing, early prediction of the adverse effects that COVID-19 infection may have on patients with FOP may have contributed to this decrease by favoring a more cautious approach to social interactions. However, the data also suggested that patients with FOP still went to see their physicians at least once during the pandemic, suggesting a strong connection to their health providers. A limitation of this study is that we do not know if these visits were telemedicine or in-person. Notably, there was a large decrease in the number of visits to a dentist. Since dental health is a major concern in FOP, encouraging patients with FOP to resume adequate care for their mental, physical, and dental health will be critical over the next several years. These findings are also important for planning post-pandemic medical management and resources for both the FOP and general communities.

Although FOP is a genetic disease characterized by progressive and severe HO and an overactive immune system, we found that most patients in our cohort with FOP and COVID-19 did not report major complications, hospitalizations, or an increase in FOP disease activity as a result of contracting COVID-19. This contrasts with recent case reports, including a 45-year-old female patient with known FOP who reported disease progression potentially linked to viral illness [35]. A month after contracting a mild form of COVID-19 infection, the patient experienced intermittent, mild abdominal pain. Two months later, computerized tomography (CT) and cytokine analysis were performed. The CT scan demonstrated HO formation in the lower abdomen and left part of the neck. In this patient, cytokine analysis revealed that 21 out of the 23 analyzed levels of cytokines were above normal levels. When combined with the current study, this suggests that patients with FOP are still at risk of complications from COVID-19, but they may tolerate exposure to SARS-CoV-2 with standard of care COVID-19 management. Our sample sizes remain too small to estimate whether this risk is significantly different from the general population.

Although intramuscular immunizations are largely avoided in patients with FOP due to the risks of complications [8, 17], we found that patients with FOP tolerated the COVID-19 vaccines well, even when given intramuscularly. Our cohort received the Pfizer-BioNTech, Moderna, CoronaVac, and Johnson & Johnson vaccines, with only one individual reporting increased FOP disease activity resembling a flare (see Case Study 4), and none reporting any serious complications or hospitalizations in the post-vaccination period. We recommended that all patients follow the International Clinical Council on FOP (ICC) guidelines for administering the injections, which included precautions with anti-inflammatory medications. Those guidelines can be found on the ICC website (http://www.iccfop.org) and are summarized in Table 3.

There are several limitations to this study. Since the reported cohort of patients with FOP who received a COVID-19 vaccine is very small, the absence of a pattern of complications or HO formation at the injection site is reassuring, but patients should be reminded that COVID-19 and any intramuscular injection still has risks in patients with FOP. Patients with FOP should still consult with their physicians before deciding to get a COVID-19 vaccine as there may be other factors that should be considered. Our study also does not provide information about the safety or efficacy of other vaccinations, so following the current treatment guidelines for patients with FOP (found at ICCFOP.org) remains important for maintaining patient safety. Notably, Subject 3 did not show IgG antibody production after receiving the first injection of the Pfizer-BioNTech vaccine subcutaneously, but did after the second dose that was given intramuscularly. While this is not proof that subcutaneous injections are ineffective, we support the ICC guidelines, which recommend that the COVID-19 vaccine be administered via the route recommended by the manufacturer, including intramuscularly, with anti-inflammatory medications given post-innoculation.

## Conclusions

Our results show that patients with FOP are at risk of decreased physical, mental, and dental health as a result of the COVID-19 pandemic. Among our limited cohort of patients with FOP, no one reported life-threatening complications with SARS-CoV-2 viral infection, although patients with FOP remain at high risk of complications from any respiratory illness because of cardiopulmonary involvement of FOP. Only one patient with FOP reported increased FOP disease activity in response to a COVID-19 vaccine; however, case reports in the literature and our own case studies suggest that patients with FOP remain at significant risk for complications such as flares and heterotopic ossification. Thus, the risks and benefits of COVID-19 vaccination needs to be discussed carefully so as to support informed decisions. Finally, our results provide a clinical foundation for future studies to understand the specific triggers of FOP-related immune responses.

## Data Availability

The data that supports the findings of this study are available from the corresponding author upon reasonable request.

## Abbreviations

ACVR1/ALK2: Activin receptor type 1A
BMP: Bone morphogenetic protein
CAJIS: Cumulative Analog Joint Involvement Scale
FOP: Fibrodysplasia ossificans progressiva
HO: Heterotopic ossification
PROMIS: Patient-reported outcomes measurement information system
IFOPA: International FOP Association
ICC: International Clinical Council on FOP
COVID: Coronavirus disease
SARS: Severe acute respiratory syndrome
